# Severe case of covid-19 infection in children: Case report

**DOI:** 10.1016/j.amsu.2021.102805

**Published:** 2021-09-05

**Authors:** M. Maarad, G. El Aidouni, A. El Kaouini, M. Aabdi, A. El Rhalet, F.Z. Aftiss, C. Bahouh, S. El Mezzeoui, H. Bkiyar, B. Housni

**Affiliations:** a Intensive Care Unit, Mohammed VI University Hospital Center, Oujda, Morocco; b Mohammed First University, Faculty of Medicine and Pharmacy, Oujda, Morocco; c Mohammed First University Oujda, FMP Oujda, LAMCESM, 60000, Oujda, Morocco

**Keywords:** Covid-19 infection, Children, Severe, Case report

## Abstract

**Introduction:**

Although covid-19 infection manifest with mild symptoms in children, it might be serious or even fatal forms of this disease.

**Case report:**

In this paper, we report three cases of severe forms of pulmonary covid-19 infection in children with several studies have shown that the mortality rate is higher in adults, mainly the elderly and the immune compromised, on the other hand a severe form in children with hospitalization in intensive care is often lethal.

**Conclusion:**

Mortality in the pediatric population with covid-19 is linked to both the severity of the pulmonary involvement and the heavy impact of intensive care.

## Highlights

• Covid-19 is a severe infection with severe complications that might lead to death.
• Children are considered are the risk group.
• In this paper, we will represent the case report of 3 children with severe outcomes (2 deaths and 1 patient survived).

## 1 Introduction

Covid-19 infection virus mainly infects elderly people with associated comorbidities [1]. The clinical manifestations of this disease are less severe in the pediatric population, often the disease goes unnoticed or typical mild symptoms cough, moderate fever, myalgia may appear [2,3].

Epidemiological data in COVID-19 viral infection in children remains rare due to a lack of published studies in this population group [4].

This study reported three severe forms of Covid-19 infection in children requiring hospitalization in a pediatric intensive care unit and contributing to the understanding to the new coronavirus in pediatric population.

## 2 Cases reports management

### 2.1 Case number 1

A 4-month-old male infant, with no notable pathological history: no consanguinity, normal childbirth, no neonatal stay, was admitted for the management of respiratory distress 10 days after an upper respiratory infection. The initial clinical evaluation: hypotonic, cyanotic infant, a heart rate of 204 bpm, a mean arterial pressure at 63 mmhg, a pulsed oxygen saturation at 75% in ambient air, a respiratory rate at 50 cycles per minute and the presence of the signs of struggles. He was hypoxemic at the arterial blood gas (PaO2 63 mmHg).

**Fig. 1:**
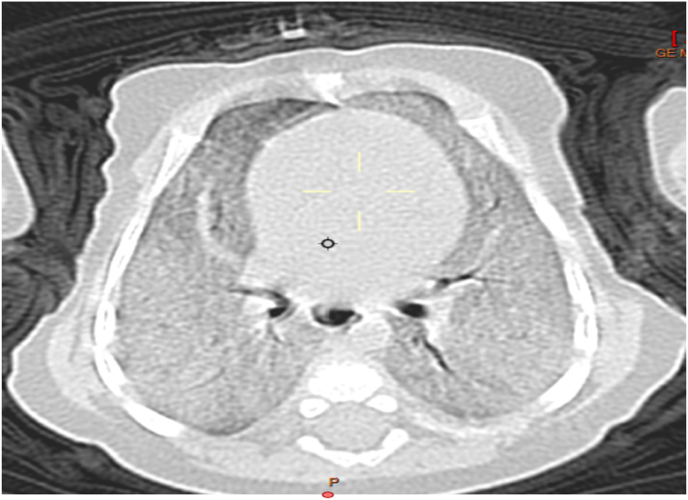
Thoracic CT scan showing lung damage of 95%.

Diagnosis of COVID-19 pneumonia was based on the positivity of the PCR and CT scan that revealed a bilateral ground-glass opacity with crazy paving pattern estimated at 95% (Fig. 1).

On the biological assessment: white blood cells (WBC) was high at 23,900 cells/mm^3^, C-reactive protein (CRP) high at 104 mg/L, lactate dehydrogenase (LDH) high at 1936 UI/L, high level of ferritin with 208,56 μg/L, normal kidney function and D-Dimers, without lymphopenia, interleukin 6 (IL6) analysis was not available at that time in our city.

He was put on oxygen therapy by mask at high concentration with a flow rate of 15 L per minute, the evolution was marked by the worsening of the respiratory distress with the appearance of hemodynamic instability requiring the use of invasive ventilation with the introduction of vasoactive drugs (Noradrenaline 0.25 mcg/kg/min). The outcome was unfavorable, the infants died after 24 hours of hospitalization in pediatric intensive care.

### 2.2 Case number 2

A five-year-old male child with no particular pathological history, admitted to our intensive care unit after 9 days of symptoms with high fever, dry cough, asthenia, diarrhea and dyspnea. Physical examination on his admission showed a deterioration of general condition with fever at 39 °C, normal hemodynamic state, respiratory rate at 42 cycles/min and his SpO2 was at 87% in ambient air. Oxygen therapy was delivered by a nasal cannula with a flow of 5 l/min.

**Fig. 2:**
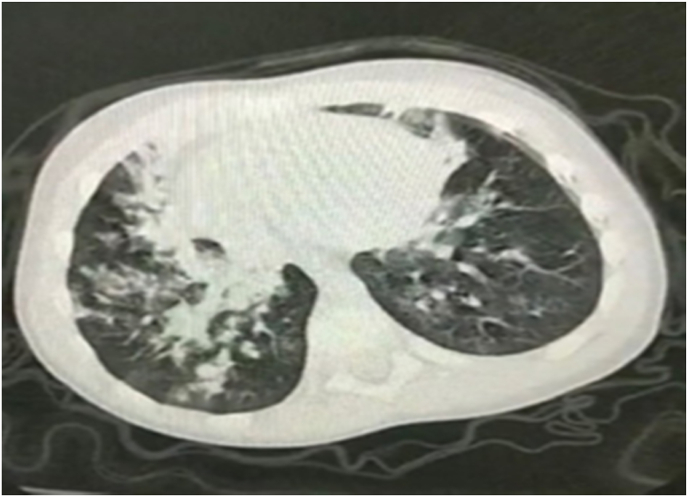
Thoracic CT scan showing lung damage of 30%.

The PCR was performed returning positive, Computed tomography (CT) scan showed bilateral ground-glass opacity of Covid-19 pneumonia estimated at 30% of lung damage (Fig. 2).

The complete blood count showed a high C-reactive protein with 45 mg/L, high level of ferritin (354 μg/L), LDH with 1342 UI/L and D-dimer, normal white blood cells with 6500 cells/mm3, normal fibrinogen, normal kidney function, without lymphopenia. IL6 analysis was not available at that time in our city.

The evolution was marked by clinical improvement under an oxygen shield. Throughout his stay in the intensive care unit, oral nutrition was maintained, as was social interaction face-to-face with loved ones. The child was transferred to pediatric department after 3 days of hospitalization in pediatric intensive care unit.

### 2.3 Case number 3

A 5-year-old female child, initially hospitalized in the pediatric department for fever and asthenia. The evolution was marked by the appearance of a dry cough, dyspnea and an 88% desaturation under 15l/min of Oxygen. The patient was transferred to our pediatric intensive care. The initial clinical assessment was as follows: Glasgow coma-scale (GCS) 15/15, fever at 39 °C, hemodynamic stability, respiratory rate at 39 cycles/min and her SpO2 at 62% in ambient air. The arterial blood gas showed a hypoxemic respiratory failure (PaO2 57).

**Fig. 3:**
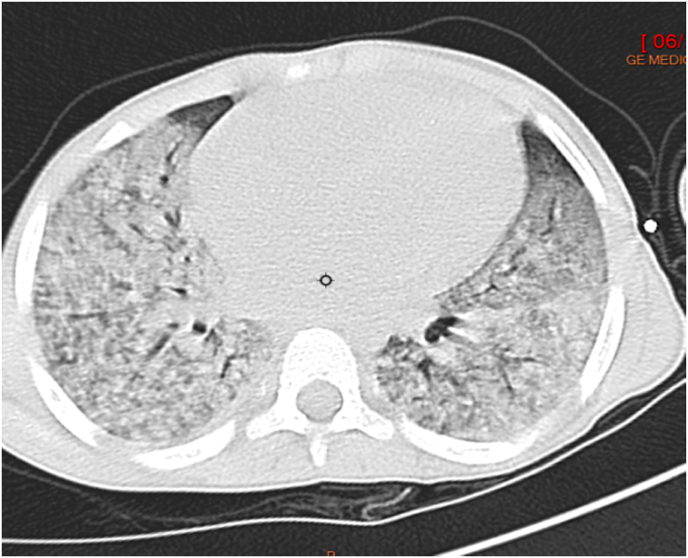
Thoracic CT scan showing lung damage of >90%.

She was diagnostic with COVID-19 infection and her Nucleic acid test was positive. The chest CT revealed a critical impairment of more than 90% (Fig. 3).

Her blood work demonstrated a high level of D-Dimer, Fibrinogen, Ferritin with 759 μg/L, the inflammatory balance was disturbed with a GB level of 20230 cells/mm3 and a high CRP level of 104 mg/l and LDH with 975 UI/l. normal kidney function was noted and without lymphopenia. IL6 analysis was not available at that time in our city.

The medical treatment was initiated by antibiotics, corticosteroids (Dexamethasone 6mg per day), and curative anticoagulation by Tinzaparin.

The course was unfavorable despite the use of mechanical ventilation and hemodynamic support. The patient died after three days of his hospitalization.

This case report follows scare guidelines [5].

## 3 Discussion

In this article, we report 3 cases of potentially severe forms of pulmonary involvement associated with covid-19 in the pediatric population.

Severe forms of Covid-19 infection in children are rare with a low mortality rate, a Chinese study published in 2020 objected to a mortality rate of around 2% in the pediatric population [6]. In the rest of the world, death associated with SARS-CoV-2 infection in children has also been reported very rarely [2,7,8].

Cai et al. reported that the median incubation period of corona virus was 7–8 days in the pediatric population [9]. The incubation period may be affected by the severity of symptoms, severe cases tended to have a shorter incubation period. A study carried out in 2003 during a similar epidemic confirms this hypothesis [10].

The rate of asymptomatic COVID-19 cases may be higher in children, in addition there may be fewer children tested for COVID-19 compared to the adult population [11].

The inflammatory assessment was high in our 2 patients who died on admission while it was normal in the surviving patient. while Carmen Lok Tung Ho and All observed that more than two-thirds (68.7%, n = 57) of cases had normal white blood cell counts, and C-reactive protein (CRP) was non-elevated in normal 70, 1% or 47/67 of patients [12].

The family accompanying our patients were satisfied with our medical care.

Our work is carried out on only severe forms of pulmonary infection linked to sars-cov2 in a pediatric population of three patients, or we note the death of two patients. Several studies have shown that the mortality rate is higher in the adult population and this may be due to associated comorbidities in adults, mainly in the elderly and the immunocompromised [13].

## 4 Conclusion

Severe forms of lung disease associated with SARS Cov-2 in children are rare. On the other hand, a severe one requiring hospitalization in intensive care and ventilator assistance, as in the case of our patients. Mortality in children with covid-19 is linked both to the severity of the pulmonary involvement, and to the length of care in pediatric intensive care.

## Ethical approval

The ethical committee approval was not required give the article type case report. However, the written consent to publish the clinical data of the patients were given and is available to check by the handling editor if needed.

## Sources of funding

This research did not receive any specific grant from funding agencies in the public, commercial, or not-for-profit sectors.

## Consent

The consent was obtained from the children's holders.

## Author contribution

MAARAD MOHAMMED: Study concept, Data collection; data analysis; writing review & editing.

EL AIDOUNI GHIZLANE: Study conception, data analysis.

EL KAOUINI ABDERRAHIM: Contributor.

AABDI MOHAMMED: Contributor.

AFTISS FATIME ZAHRA: Contributor.

BAHOUH CHOUKRI: Contributor.

EL MEZZEOUI SANAE: Contributor.

EL RHALET ABDELILAH: Contributor.

BKIYAH HOUSSAM: Supervision and data validation.

HOUSNI BRAHIM: Supervision and data validation.

## Registration of research studies

This is not an original research project involving human participants in an interventional or an observational study but a case report. This registration was not required.

## Guarantor

MAARAD Mohammed.

## Provenance and peer review

Not commissioned, externally peer-reviewed.

## Declaration of competing interest

The authors state that they have no conflicts of interest for this report.
